# Tensor-cell2cell v2 unravels coordinated dynamics of protein- and metabolite-mediated cell–cell communication

**DOI:** 10.1093/bioinformatics/btaf667

**Published:** 2026-02-20

**Authors:** Erick Armingol, Reid O Larsen, Lia Gale, Martin Cequeira, Hratch M Baghdassarian, Nathan E Lewis

**Affiliations:** Bioinformatics and Systems Biology Graduate Program, University of California San Diego, La Jolla, CA 92093, United States; Biomedical Sciences Graduate Program, University of California San Diego, La Jolla, CA 92093, United States; Department of Pharmacology, University of California San Diego, La Jolla, CA 92093, United States; Bioinformatics and Systems Biology Graduate Program, University of California San Diego, La Jolla, CA 92093, United States; Center for Molecular Medicine, Complex Carbohydrate Research Center, and Department of Biochemistry and Molecular Biology, University of Georgia, Athens, GA 30602, United States; Bioinformatics and Systems Biology Graduate Program, University of California San Diego, La Jolla, CA 92093, United States; Center for Molecular Medicine, Complex Carbohydrate Research Center, and Department of Biochemistry and Molecular Biology, University of Georgia, Athens, GA 30602, United States; Department of Pediatrics, University of California San Diego, La Jolla, CA 92093, United States; Department of Bioengineering, University of California San Diego, La Jolla, CA 92093, United States

## Abstract

**Summary:**

Cell–cell communication dynamically changes across time while involving diverse cell populations and ligand types such as proteins and metabolites. Single-cell transcriptomics enables its inference, but existing tools typically analyze ligand types separately and overlook their coordinated activity. Here, we present Tensor-cell2cell v2, a computational tool that can jointly analyze protein- and metabolite-mediated communication over time using coupled tensor component analysis, while preserving each modality of inferred communication scores independently, as well as their data structures and distributions. Applied to brain organoid development, Tensor-cell2cell v2 uncovers dynamic, coordinated communication programs involving key proteins and metabolites across relevant cell types and specific time points.

**Availability and implementation:**

Tensor-cell2cell v2 and its new coupled tensor component analysis are implemented in Python and available as part of the cell2cell framework at https://github.com/earmingol/cell2cell. This python library is available on PyPI. Code for the analyses of this manuscript can be found in a Code Ocean capsule at https://doi.org/10.24433/CO.0061424.v3, where analyses can be also run and reproduced online. Tutorials can be found at https://cell2cell.readthedocs.io.

## 1 Introduction

Cell–cell communication (CCC) is multimodal and dynamic, involving both small molecule and protein signals that act with fine spatiotemporal coordination to drive multicellular functions (Armingol *et al.* 2021). This synchronization arises through mechanisms such as transcription factors (TFs) controlling the production of multiple ligands, receptors with shared functions, and phosphorylase/phosphatase systems modulating signaling cascades (Baker and Rutter 2023, Hornisch and Piazza 2025). Although single-cell transcriptomics has enabled computational methods to infer cell–cell communication from gene expression (Almet *et al.* 2021, Armingol *et al.* 2024, Cesaro *et al.* 2025), most of them are focused on protein-based ligand–receptor (LR) interactions.

Recent efforts incorporate metabolite ligands for inferring CCC from single-cell transcriptomics (Dimitrov *et al.* 2024, Zhang *et al.* 2024, Troulé *et al.* 2025, Zheng *et al.* 2025). However, they do not account for both protein- and metabolite-mediated CCC simultaneously, often evaluating them separately. Protein-mediated CCC can be directly inferred from gene expression, whereas metabolite-mediated CCC is inferred indirectly from enzymes that produce or consume small molecules (Zheng *et al.* 2025). Consequently, protein- and metabolite-based scores are only informative in isolation as they differ in nature and scale, limiting their joint interpretation. Moreover, existing tools do not consider temporal coordination of LR pairs, preventing insights of protein and metabolite ligands acting in concert.

Coupled factorization approaches linking tensors and/or matrices have demonstrated utility in revealing coordinated patterns across multimodal biological datasets while preserving the inherent data structure of each modality (Tan and Meyer 2024). For example, such approaches have been used to jointly analyze static and dynamic metabolomics data, revealing shared subject patterns linked to biomarker profiles from both modalities (Li *et al.* 2024). To find dynamic CCC patterns driven by biological contexts, we previously introduced Tensor-cell2cell, a tool that applies tensor component analysis (TCA) on one modality of CCC and enables the incorporation of time as the biological context (Armingol *et al.* 2022a). Expanding this idea, coupled tensor factorization is particularly suited for studying multimodal CCC, while also considering shared changes across modalities in relation to cellular contexts (e.g. time points). Thus, coupled tensor component analysis (CTCA) provides a powerful strategy to integrate protein- and metabolite-mediated signals, capturing shared dynamics while preserving the distinct score distributions and biological insights of each modality.

Here, we present Tensor-cell2cell v2, a new version of our tool enabling CTCA to perform a joint evaluation of protein and metabolite LR activity across time while maintaining each modality’s data structure and score distribution. We applied it to cortical brain organoid data, uncovering time-driven communication programs coordinating both ligand types during cortical development.

## 2 Methods

### 2.1 Preprocessing of the RNA-seq data

**Figure 1.**
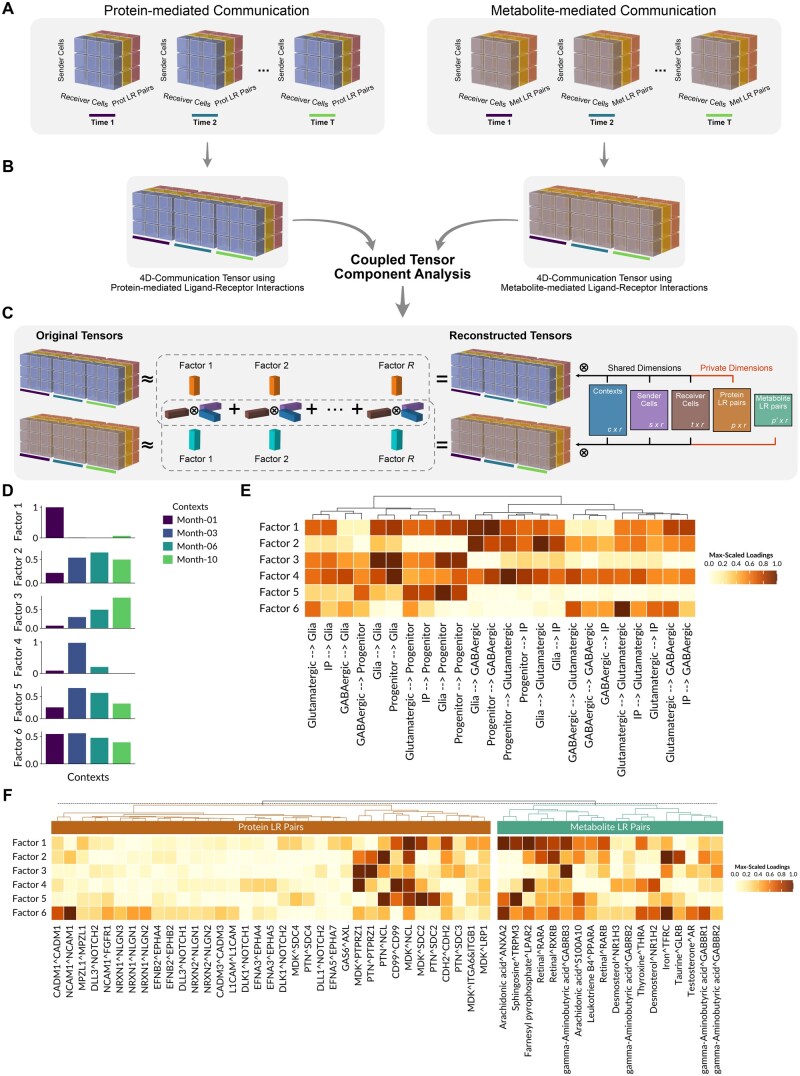
CTCA for detecting coordinated protein- and metabolite-based communication patterns. (A) 4D tensors are built separately for protein- and metabolite-mediated communication modalities by computing communication scores for each LR pair in every combination of sender-receiver cell pairs at each time point, using cell2cell and MEBOCOST, respectively. (B) These tensors are jointly analyzed with our non-negative coupled tensor component analysis (CTCA). (C) CTCA decomposes both tensors into *R* factors, each of which is the outer product (⊗) among loadings vectors (colored boxes) of the shared and private tensor dimensions. Shared dimensions are contexts, sender cells, and receiver cells, and their loading vectors (blue, purple, and brown boxes) couple both modalities, while loading vectors for private dimensions (orange and cyan boxes) capture protein and metabolite-specific LR pairs. Summing these factors reconstructs each tensor. These tensors can also be represented as outer products of dimension-specific matrices (colored rectangles), each containing loadings for the corresponding dimension elements (rows) across factors (columns), as indicated by their size at their bottom right corner. (D–F) Application of CTCA to cortical brain organoid scRNA-seq data reveals six CCC programs (factors). (D) Context loadings per factors reflect shared dynamics of CCC between modalities. (E) Heatmap of sender-receiver cell pair loadings per factor, hierarchically clustered. These joint loadings were computed as the outer product of the shared loadings of sender and receiver cell dimensions. (F) Heatmaps of protein- and metabolite-specific LR loadings per factor, hierarchically clustered separately by ligand type. Only LR pairs with high loadings in at least one factor are displayed. Co-occurrence of high-loading protein and metabolite LR pairs within the same factor reflects their coordinated communication dynamics. Loadings in (E, F) were normalized per factor to the maximum within each modality to highlight important features. IP, intermediate progenitor; LR, ligand–receptor.

We analyzed single-cell RNA-seq data from cortical brain organoids at 1, 3, 6, and 10 months (Trujillo *et al.* 2019) available under GEO accession number GSE130238. To reconstruct the original study annotations based on pertinent markers, we processed this data as indicated in the Section 1, available as supplementary data at *Bioinformatics* online and Fig. 1, available as supplementary data at *Bioinformatics* online.

### 2.2 Inferring cell–cell communication

Normalized and log-transformed gene expression (log1p(CP10k) values) was used as input for cell2cell (Armingol *et al.* 2022b) and MEBOCOST (Zheng *et al.* 2025) to infer protein- and metabolite-mediated CCC, respectively. For protein-mediated CCC, we used CellChatDB (Jin *et al.* 2021), which contains 2005 human protein-based LR pairs; for metabolite-mediated CCC, we used the MEBOCOST database (Zheng *et al.* 2025), which includes 782 metabolite-based ligand-sensor interactions. For each modality, we constructed a 4D communication tensor from the resulting scores, organized by time points, LR pairs, and sender and receiver cell types (Armingol *et al.* 2022a).

### 2.3 Non-negative coupled tensor component analysis

Building upon a non-negative TCA (Williams *et al.* 2018), we implemented a non-negative CTCA to simultaneously factorize two non-negative tensors sharing all but one mode (i.e. dimension). To detail this approach, let χ and χ′ represent two tensors of size *C* × *P* × *S* × *T* and *C* × *P′* × *S* × *T*, respectively, where *C*, *S* and *T* correspond to the shared number of contexts/samples, sender cells and receiver cells, while *P* and *P’* correspond to the tensor-specific number of LR pairs (or any other private dimension), representing protein- and metabolite-based LR pairs, respectively. Hence, $\chi_{\mathrm{ijkl}}$ and ${\chi \prime }_{\mathrm{imkl}}$ denote the representative interactions of context *i*, using the protein- and metabolite-based LR pairs *j* and *m* respectively, between the sender cell *k* and receiver cell *l*.

The CTCA method corresponds to a coupled version of the CANDECOMP/PARAFAC decomposition (Carroll and Chang 1970, Harshman *et al.* 1970), yielding the simultaneous factorization of χ and χ′, each through a sum of *R* tensors of rank-1:


(1a) $$\chi \;\approx \sum_{r\;=\;1}^{R}c^{r}⊗p^{r}⊗s^{r}⊗t^{r}$$

(1b) $$\chi \prime \;\approx \sum_{r\;=\;1}^{R}c^{r}⊗{p\prime }^{r}⊗s^{r}⊗t^{r}$$

where the notation ⊗ represents the outer product and $c^{r},s^{r}and\;t^{r}$ are shared vectors across both tensors, while $p^{r}\;$and ${p\prime }^{r}$ are tensor-specific vectors for the private dimension (here, LR pairs). Each rank-1 tensor corresponds to a factor *r* with non-negative entries. Similar to non-negative matrix factorization (NMF), these factors are interchangeable, and elements with higher loadings within a dimension indicate stronger contributions to the biological pattern captured by that factor. These factors are further used to describe an element with indices *ijkl* in each of the original tensors, which can be approximated as the sum of all elements with same indices across rank-1 tensors (factors):


(2a) $$\chi_{\mathrm{ijkl}}\;\approx \sum_{r\;=\;1}^{R}c_{i}^{r}{\times \;p}_{j}^{r}\;\times {\;s}_{k}^{r}\times \;t_{l}^{r}$$

(2b) $${\chi \prime }_{\mathrm{imkl}}\;\approx \sum_{r\;=\;1}^{R}c_{i}^{r}{\;\times \;p\prime }_{m}^{r}\;\times \;s_{k}^{r}\times \;t_{l}^{r}$$

The coupled tensor factorization is performed by iterating the following objective function until convergence through an alternating least squares minimization (Anandkumar *et al.* 2014, Rabanser *et al.* 2017):


(3) $$\min_{\left\{c,\;p,\;p\prime ,\;s,\;t\right\}}\;{\propto_{1}|\left|M*(\chi \;-\sum_{r\;=\;1}^{R}c^{r}⊗p^{r}⊗s^{r}⊗t^{r})\right||}_{F}^{2}+{\propto_{2}|\left|M\prime *(\chi \prime \;-\sum_{r\;=\;1}^{R}c^{r}⊗{p'}^{r}⊗s^{r}⊗t^{r})\right||}_{F}^{2}$$

where ${\left|\left|\;.\;\right|\right|}_{F}^{2}$ represents the squared Frobenius norm, and $\propto_{1}$ and $\propto_{2}$ are balancing weights that sum up to one. These weights control how each tensor contributes to the factorization results. These balancing weights are controllable parameters in the CTCA, which can be changed by the user upon reasonable criteria to influence the discovered patterns. Alternatively, it can be used as implemented in other coupled factorization approaches (Li *et al.* 2024), where ${\propto_{1}=\propto }_{2}=0.5$.

To handle missing data, we adopted a previous strategy of using masked values (Williams *et al.* 2018): *M* and *M′* are two tensors of size *C* × *P* × *S* × *T* and *C* x *P′* × *S* × *T*, respectively, where entries $M_{\mathrm{ijkl}}$ and ${M\prime }_{\mathrm{imkl}}$ equal zero to indicate missing values for the corresponding entries in χ and χ′, and equal one to indicate the opposite. When no missing values are present or they are assumed to be real zeros, all entries in *M* and *M’* should equal one. In Equation (3), ∗ denotes element-wise multiplication (Hadamard product) of two tensors.

A detailed implementation of the CTCA algorithm and metrics to evaluate its performance can be found in the Supplementary Section S2, available as supplementary data at *Bioinformatics* online. Additionally, analyses for selecting the number of factors and assessing the robustness of the CTCA results can be found in the Supplementary Section S3, available as supplementary data at *Bioinformatics* online.

## 3 Results

We extended the functionalities of Tensor-cell2cell, a TCA framework for identifying context-driven communication programs, to investigate how CCC mediated by proteins and metabolites is coordinated. Briefly, Tensor-cell2cell summarizes each communication pattern through latent factors that capture combinations of sender-receiver cell pairs and their signaling molecules, while linking them to specific dynamics across biological contexts. In this second version, we implemented a non-negative CTCA devised to jointly analyze protein- and metabolite-mediated CCC across time (Fig. 1A–C), overcoming the limitations of existing approaches that analyze these modalities separately and do not account for a temporal dimension.

To illustrate the utility of the CTCA, we applied it on a dataset of cortical brain organoids containing multiple time points that represent different stages of cortical brain development (Trujillo *et al.* 2019). In this complex process, neural and non-neural cell types must be produced in the correct number by following appropriate spatiotemporal dynamics of CCC (Jiang and Nardelli 2016). After building both coupled tensors for this dataset (Fig. 1A–C) and identifying an appropriate number of factors (Supplementary Fig. S2, available as supplementary data at *Bioinformatics* online), we used Tensor-cell2cell v2 to detect six CCC programs coordinating both ligand types (factors in Fig. 1D–F). These programs capture distinct dynamics during the development of cortical brain organoids, with spikes in specific time points (factors 1 and 4) or gradual changes across time points (factors 2, 3, 5, and 6) (Fig. 1D).

The CTCA is key to identify coordination from two modalities across specific temporal dynamics and sender-receiver cells. Overall, it tends to capture CCC programs enriched by protein and metabolite ligands sharing transcriptional regulation as well as receptors from both modalities with similar biological functions (Supplementary Section S4, available as supplementary data at *Bioinformatics* online). Among specific CCC patterns, factor 5 was dominated by communication from different sender cells to progenitors and to a lesser extent to glial cells as receivers (Fig. 1E), reflecting a progenitor-centered communication hub. Indeed, it was characterized by progenitor-supporting signals, including CD99–CD99 self-signaling, MDK–SDC2 and PTN–SDC2 growth factor interactions, and DLL3/DLK1–NOTCH2 in the protein modality, acting in concert with metabolite LR pairs such as iron–TFRC, arachidonic acid–ANXA2/S100A10, sphingosine–TRPM3, and retinal–RARA/RXRB (Fig. 1F). Remarkably, the presence of arachidonic acid has been demonstrated to influence MDK signaling (Yao *et al.* 2025). Moreover, these results are consistent with programs that sustain and regulate progenitor populations (Zou *et al.* 2006, Borghese *et al.* 2010, Sakayori *et al.* 2011). In contrast, factor 6 was defined by signaling mainly involving glutamatergic and GABAergic neurons (Fig. 1E), indicating a neuronal-centered program rather than progenitor-focused communication. This CCC program was characterized by neuronal adhesion and synaptic LR pairs (e.g. L1CAM, NCAM1 and CADM1 self-interactions, Ephrins interactions with their cognate receptors, and Neurexin–Neuroligin interactions) together with metabolites such as GABA, retinoids, testosterone, and thyroxine (Fig. 1F), which are key in neuronal-centered programs including migration, differentiation, and circuit-building (Bernal 2007, Maden 2007, Wu and Sun 2015, Kelava *et al.* 2022). Thus, our results highlight how CTCA reveals the coordinated action of diverse molecular signals that guide progenitor maintenance and neuronal maturation.

## 4 Discussion

We demonstrate through our implementation of a non-negative CTCA that Tensor-cell2cell v2 can decipher the coordinated temporal dynamics of both protein- and metabolite-mediated CCC from single-cell omics. Unlike existing methods, it jointly analyzes modalities with distinct behaviors, such as protein-based communication (directly predicted from gene expression) and metabolite-based communication (indirectly inferred from enzyme expression). Tensor-cell2cell builds modality-specific 4D communication tensors and couples them through CTCA, aligning their dynamics across contexts and sender-receiver cell pairs, which would otherwise show mismatched behaviors if analyzed separately. This facilitates the identification of key LR pairs across ligand types acting in concert within pertinent CCC programs.

Applied to cortical brain organoid development (Trujillo *et al.* 2019), our approach revealed concerted dynamics of specific protein and metabolite ligands. For example, both MDK and retinal interactions with their cognate receptors were repeatedly captured across factors (Fig. 1F). MDK is encoded by a retinoic acid (RA)-responsive gene (Kadomatsu and Muramatsu 2004), and retinal is the precursor of RA, a key developmental regulator (Maden 2007). CTCA also identified progenitor-centered programs involving Notch and arachidonic acid signaling (factor 5 in Fig. 1F), whose coordinated action helps maintain a proliferative state instead of undergoing differentiation (Sakayori *et al.* 2011, Ohtaka-Maruyama and Okado 2015, Jiang and Nardelli 2016). These findings illustrate how Tensor-cell2cell v2 uncovers biologically meaningful coordination between protein- and metabolite-mediated communication, which can arise when the same TFs regulate the production of both the protein and metabolite ligands (Supplementary Figs S3-S5, available as supplementary data at *Bioinformatics* online) or when their corresponding receptors participate in shared biological processes (Supplementary Fig. S6, available as supplementary data at *Bioinformatics* online).

Altogether, Tensor-cell2cell v2 provides a flexible framework to integrate multiple CCC modalities. Although we used cell2cell (Armingol *et al.* 2022b) and MEBOCOST (Zheng *et al.* 2025), users can adopt alternative tools for each modality, depending on their needs and each tool’s underlying assumptions. Beyond proteins and metabolites, CTCA can couple classical communication scores (Armingol *et al.* 2022a) with also other diverse modalities, such as extracellular vesicle signaling (Shao *et al.* 2025), transcription factor activities (Badia-I-Mompel *et al.* 2022), or intracellular metabolism (Armingol *et al.* 2025). By harmonizing heterogeneous scores into multimodal CCC programs, Tensor-cell2cell v2 facilitates the discovery of coordinated molecular mediators shaping cellular contexts, and enables future multimodal analyses.

## Supplementary Material

btaf667_Supplementary_Data

## Data Availability

The data underlying this article are available in Code Ocean at https://doi.org/10.24433/CO.0061424.v3. Additionally, raw single-cell RNA-seq data from cortical brain organoids at 1, 3, 6, and 10 months are available in Gene Expression Omnibus (GEO) at https://www.ncbi.nlm.nih.gov/geo, and can be accesed with accession number GSE130238.
