# Supplementary material for: Tensor-cell2cell v2 unravels coordinated dynamics of protein- and metabolite-mediated cell–cell communication
Source: Bioinformatics. 2026 Feb 20;42(2):btaf667. doi: 10.1093/bioinformatics/btaf667 (PMC12937581; doi:10.1093/bioinformatics/btaf667)
Supplement: btaf667_Supplementary_Data [file btaf667_supplementary_data.pdf]

Supplementary Material for

Tensor-cell2cell v2 unravels coordinated dynamics of protein- and metabolite-mediated cell-cell communication

Erick Armingol[1*], Reid O. Larsen[2,3], Lia Gale[1,4], Martin Cequeira, Hratch M. Baghdassarian[1], Nathan E. Lewis[4,5,6*]

[1] Bioinformatics and Systems Biology Graduate Program, University of California San Diego, La Jolla, CA 92093, United States
[2] Biomedical Sciences Graduate Program, University of California San Diego, La Jolla, CA 92093, United States
[3] Department of Pharmacology, University of California San Diego, La Jolla, CA 92093, United States
[4] Center for Molecular Medicine, Complex Carbohydrate Research Center, and Department of Biochemistry and Molecular Biology, University of Georgia, Athens, GA 30602, United States
[5] Department of Pediatrics, University of California San Diego, La Jolla, CA 92093, United States
[6] Department of Bioengineering, University of California San Diego, La Jolla, CA 92093, United States

*Correspondence: Erick Armingol. E-mail: erickarmingol@gmail.com; Nathan E. Lewis. E-mail: natelewis@uga.edu. Center for Molecular Medicine, University of Georgia, 325 Riverbend Rd, Athens, GA 30602, United States.

Contents:

- **Supplementary Section S1: Clustering and annotation of the single-cell data of cortical organoids.**
  - Supplementary Figure S1. Annotation of the single-cell dataset of the cortical organoids.
- **Supplementary Section S2: Implementation of the CTCA algorithm and metrics.**
- **Supplementary Section S3: Evaluations of the CTCA on the single-cell data of cortical organoids.**
  - Supplementary Figure S2. Evaluation of CTCA performance, parameter selection, and robustness on single-cell cortical organoid data.
- **Supplementary Section S4: Biological evidence of potential coordination between protein and metabolite ligands.**
  - Supplementary Table S1. Enrichment analysis of protein-metabolite ligand pairs controlled by shared transcription factors.
  - Supplementary Figure S3. Regulatory network connecting transcription factors with protein and metabolite ligands.
  - Supplementary Figure S4. Main transcription factor co-regulating protein and metabolite synthesis.
  - Supplementary Figure S5. Factor-specific regulatory networks resulting from the CTCA.
  - Supplementary Figure S6. Gene Set Enrichment Analysis suggests coordinated biological processes across protein and metabolite communication modalities.

Supplementary Section S1: Clustering and annotation of the single-cell data of cortical organoids

The original study annotations were reconstructed by processing this data with Seurat v3.0 (Stuart *et al.*, 2019). Genes detected in fewer than 3 cells per time point were removed. After merging time points, we retained cells with 200-6000 genes and <7.5% mitochondrial content. Data were normalized, log-transformed, and scaled. Principal component analysis (PCA) was performed from highly variable genes identified with the FindVariableFeatures function. Batch correction was done using harmony with default parameters (Korsunsky *et al.*, 2019). Cells were clustered using the top 20 harmony components (resolution = 0.5), yielding 17 clusters, which we merged to reconstitute the 7 cell types in the original study: glutamatergic, GABAergic, glia, progenitor, intermediate progenitor (IP), mitotic, and other cells (Supplementary Fig. S1). Similar to that work, mitotic and other cells were excluded from further analysis.

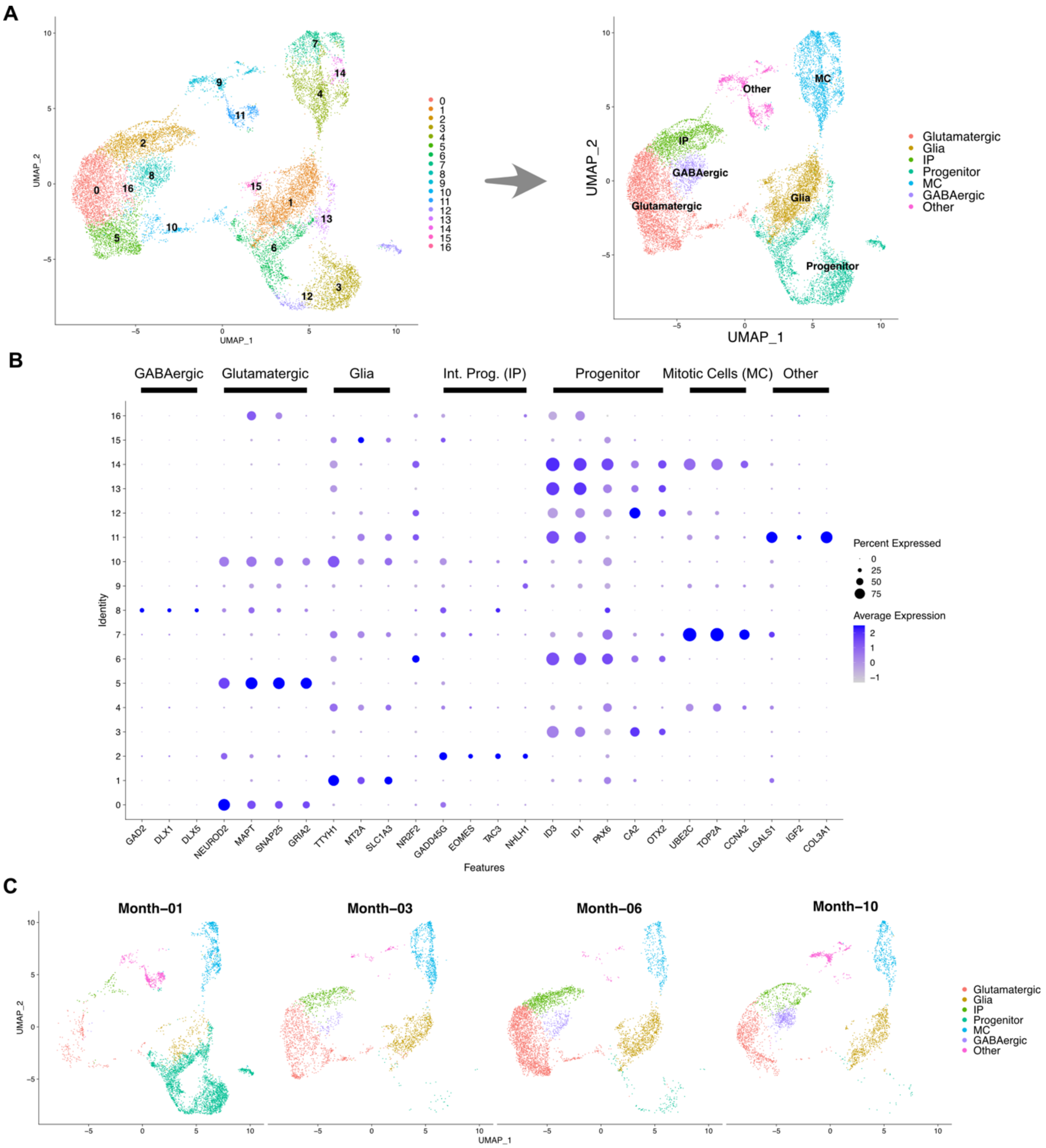

Supplementary Figure S1. Annotation of the single-cell dataset of the cortical organoids. After preprocessing the dataset and performing the cell clustering as indicated in Methods, each of the clusters was annotated according to the original annotations of cell types in this dataset (Trujillo *et al.*, 2019). (**A**) UMAP visualization of the resulting clusters and their annotations. (**B**) Detail of the

marker expression levels employed to perform the annotation. These markers were previously described by (Trujillo *et al.*, 2019). (**C**) UMAP visualization of the distinct cell types across the different time points in the dataset.

Supplementary Section S2: Implementation of the CTCA algorithm and metrics

S2.1 Algorithm for performing the CTCA.

The CANDECOMP/PARAFAC decomposition represents each tensor as a collection of factor matrices, with one factor matrix per tensor mode or dimension. For our tensors χ and χ', the CTCA results in factor matrices C, P, S, T for the first tensor and C, P', S, T for the second tensor. Each factor matrix has a shape of the number of elements in the mode x *R*, where *R* is the rank of the decomposition. For example, factor matrix C has a shape of the number of contexts x *R*, containing *R* column vectors that represent how each context contributes to each of the *R* factors. The shared factor matrices C, S, T are constrained to be identical between both tensors, while the private factor matrices P and P' are tensor specific.

The optimization procedure of our CTCA is based on an alternating least squares approach with multiplicative updates (Rabanser *et al.*, 2017). The algorithm begins by initializing the factor matrices for both tensors. We employ singular value decomposition (SVD)-based initialization, which computes an initial estimate for each factor matrix by performing a truncated SVD on the unfolded (matricized) tensor along each mode. This provides a more stable starting point that typically leads to faster convergence compared to random initialization. However, when missing data are present in the tensors (indicated by mask tensors *M* and *M'* in the equation (3) in Methods), random initialization is used instead to avoid biases that could arise from the SVD computation on incomplete data. For shared modes, the initial factor matrices are set to be identical between both tensors, ensuring coupling from the start of the optimization.

The core of the optimization consists of iteratively updating each factor matrix while holding all other factor matrices fixed. For private modes (in this case, the ligand-receptor pair dimension with factor matrices P and P' for tensors χ and χ', respectively), each tensor's factor matrix is updated independently using only information from factor matrices that are present in its respective modality. The update for factor matrix P of tensor χ takes the form:

$$P^{t+1} = P^{t} \odot (N \oslash D) \qquad \text{(S1)}$$

where $\odot$ denotes element-wise multiplication, $\oslash$ denotes element-wise division, the superscript (t) indicates the current iteration, *N* represents the numerator computed as the matricized tensor times Khatri-Rao product, and *D* represents the denominator computed from the element-wise (Hadamard) product of the Gram matrices of all other factor matrices, providing normalization. The Gram matrix of a factor matrix *F* is computed as $F^{T}F$, representing the inner products between all pairs of its columns (pairs of factors). To ensure non-negative values, *N* and *D* are clipped to a minimum value of machine epsilon, where ε depends on the floating-point precision of the data (typically about 10^{-16} for double precision). The same update rule applies independently to *P'* for tensor χ'.

For shared modes (contexts represented by C, sender cells by S, and receiver cells by T), the updates must incorporate information from both tensors simultaneously to maintain coupling. The algorithm computes separate update terms from each tensor, then combines these using the balancing weights $\propto_1$ and $\propto_2$ described in equation (3) (Methods). For a shared factor matrix S (representing sender cells), the update becomes:

$$S^{t+1} = S^{t} \odot ((\propto_1 N_1 + \propto_2 N_2) \oslash (\propto_1 D_1 + \propto_2 D_2)) \qquad \text{(S2)}$$

where N_1, D_1 are the numerator and denominator terms computed from χ, while N_2, D_2 are the corresponding terms from χ'. This weighted combination ensures that both tensors contribute to shaping the shared factors, with the relative influence controlled by the balancing weights. After computing the combined update, the same new factor matrix is assigned to the corresponding mode in both tensors, thus enforcing the coupling constraint. The same procedure applies to the other shared factors C (contexts) and T (receiver cells).

The update strategy proceeds in the following order: all tensor-specific (private) modes are updated independently for each tensor. Then, shared modes are updated, with each shared factor matrix updated once per iteration using the combined information from both tensors. This ordering helps to obtain biologically meaningful results by updating the private factors first, ensuring to find properties of each modality, which will constrain the behavior of the shared factors after (through the *N* and *D* terms).

Convergence is assessed by monitoring the reconstruction error (Supplementary Section S2.2) after each iteration. The algorithm terminates when the absolute decrease in combined error between consecutive iterations falls below a specified tolerance threshold (value of 10^{-8} in this case), indicating that further iterations provide negligible improvement. Alternatively, if convergence is not achieved, the algorithm stops after reaching a maximum number of iterations (500 iterations in this case). Upon termination, a final normalization step is applied to produce the returned factor matrices and weights.

To mitigate the risk of convergence to suboptimal local minima, our CTCA is run independently 100 times, each with a different random seed for initialization. After completing all runs, we select the solution that achieves the lowest combined reconstruction error. This strategy, combined with SVD-based initialization when no missing data are present, helps ensure convergence to more stable and reproducible optimization solutions.

S2.2 Measuring the error of the CTCA

To quantify the error of the CTCA in representing the original 4D communication tensors (χ and χ') through the lower rank tensors (sum of *R* tensors of rank-1 in equations (1a) and (1b) in Methods), we use a combined normalized reconstruction error. First, individual normalized errors are computed for each tensor:

$$E_1 = \frac{\left|\left|M*(\chi - \sum_{r=1}^{R} c^r \otimes p^r \otimes s^r \otimes t^r)\right|\right|_F^2}{||\, M*\ \chi\, ||_F^2} \tag{S3a}$$

$$E_2 = \frac{\left|\left|M'*(\chi' - \sum_{r=1}^{R} c^r \otimes p'^r \otimes s^r \otimes t^r)\right|\right|_F^2}{||M'*\ \chi'\, ||_F^2} \tag{S3b}$$

Then, the combined error *E* is calculated as a weighted average:

$$E = \propto_1 E_1 + \propto_2 E_2 \tag{S4}$$

Where *M* and *M'* are tensor masks for missing data, and $\propto_1$ and $\propto_2$ represent the balancing weights, all of them described in equation (3) (Methods). These balancing weights ensure both tensors contribute accordingly to the combined error metric. This combined error (*E*) maintains the interpretability of the standard normalized error, acting analogously to the fraction of unexplained variance (Williams *et al.*, 2018).

S2.3 Evaluating consistency and reproducibility of CTCA results

To evaluate the consistency and reproducibility of tensor decomposition results across independent runs, we employed the CorrIndex (Sobhani *et al.*, 2022). This metric was previously used in Tensor-cell2cell v1 (Armingol *et al.*, 2022) to evaluate factorization similarity, and it has been further adopted by other works through its implementation in TensorLy (Kossaifi *et al.*, 2016), a tensor factorization framework in Python. Briefly, the CorrIndex is a permutation- and scale-invariant distance metric that enables direct comparison of decompositions between tensors containing the same elements, without requiring prior alignment of the resulting factors. This is particularly important, as independent tensor factorizations can produce equivalent components in different orders or with varying scales.

We computed the CorrIndex separately for each tensor in the coupled factorization, using their corresponding modes, as well as jointly by stacking the shared and private modes to evaluate the overall consistency of the coupled decomposition. The CorrIndex ranges from 0 to 1, with higher values indicating greater dissimilarity between decomposition outputs. For clarity of interpretation, we report similarity as (1 - CorrIndex), such that higher values correspond to more consistent results across runs.

Supplementary Section S3: Evaluations of the CTCA on the single-cell data of cortical organoids

S3.1 Selection of the number of factors

To determine an appropriate number of factors (rank), we combined two complementary evaluation strategies: (i) an analysis of the similarity of solutions across multiple independent runs per rank and (ii) of the reconstruction error across varying ranks.

For each candidate rank, we performed 20 independent CTCA runs and computed both the average reproducibility of the resulting factorizations using the (1 - CorrIndex) similarity metric and the average reconstruction error. We prioritized ranks that achieved high reproducibility (similarity of at least 0.9) while maintaining low reconstruction error (Supplementary Fig. S2A). This combined criterion helps to select an adequate number of factors that lead to stable solutions while keeping a model with low error.

S3.2 Influence of the balancing weights

We implemented a weighting parameter that allows adjusting the relative contribution of each tensor to the CTCA. This parameter corresponds to the balancing weights in equation (3) (Methods), which users can adjust arbitrarily, in an informed way, or just leave it in the default value of equal contribution, as normally done by other coupled factorization methods such as in (Li *et al.*, 2024). To illustrate how this parameter can be adjusted in an informed manner, we evaluated how the resulting factorizations change under varying these weights. To measure the similarity between decompositions with different balancing weights, we employed the CorrIndex (see Supplementary Section S2.3).

The analyses revealed that different weighting configurations produced similar factorization results (Supplementary Fig. S2B), with a mild decrease when assigning a high weight to the metabolite-based tensor. To select the balancing weights in our case, we picked the combination that overall had more similarities with the rest of combinations (Supplementary Fig. S2B), corresponding to values $\alpha_1=0.7$; $\alpha_2=0.3$, which moderately prioritizes the protein modality rather than the metabolite modality.

S3.3 Definitive CTCA and its loss across iterations

Once we select the combination of number of factors and balancing weights, we carry out the tensor factorization by performing 100 independent runs with differing random seeds of initialization. From these runs, we then select the best model across them (these 100 runs and the subsequent model selection are hereinafter referred to as a "full run" of the CTCA). In this best model, the loss can be tracked across iterations of the alternating least squares approach with multiplicative updates (Supplementary Fig. S2C).

S3.4 Stability and reproducibility of the factorization

To maximize the stability and reproducibility of our CTCA results, we implemented important measures: (i) SVD-based initialization to provide a robust starting point, and (ii) running the CTCA 100 times with different random seeds, selecting the factorization with the lowest reconstruction error (i.e., a full run CTCA).

To evaluate the robustness of the CTCA results after implementing these measures, we evaluated two cases that can lead to different results due to local minima: (i) Initialization using different random seeds (i.e. different starting values of the factors), and (ii) tensors with same data but with elements ordered differently per mode.

In the first scenario, we performed 10 independent full runs of the CTCA, each of them aimed to maximize stability (100 runs with unique random seeds followed by selection of the best model). We made sure that the 1,000 total runs presented no overlap in random seeds. We then evaluated the similarity between these 10 independently selected best models, which lead to highly similar solutions, with an average (1 - CorrIndex) similarity metric above 0.99, indicating near-identical decompositions across independent runs (Supplementary Fig. S2D).

In the second case, we permuted the order of elements per mode separately. Specifically, we did five independent permutations per mode; given that there are three shared and two private modes, this resulted in 25 full runs of the CTCA, totalling 2,500 simple runs (each of the 25 full runs included 100 independent simple runs and best model selection). We limited this analysis to five permutations per mode because of the computationally-demanding nature of the analysis. Even under these permutations, the resulting factorizations remained highly similar (Supplementary Fig. S2E), with an average similarity score above 0.99.

Together, these results demonstrate that our CTCA yields highly reproducible and stable factorizations across independent initializations driven by random seeds and ordering of the input data. Moreover, when evaluating robustness by using simple runs instead of full runs, there is a decrease in the factorization similarities (Supplementary Figs 2F,G) to values ranging between 0.9 and 1. Thus, this indicates that our implementation when including full runs effectively mitigates the influence of local minima and produces consistent results for the analysis of multimodal CCC, with an average similarity above 0.99 (1 - CorrIndex).

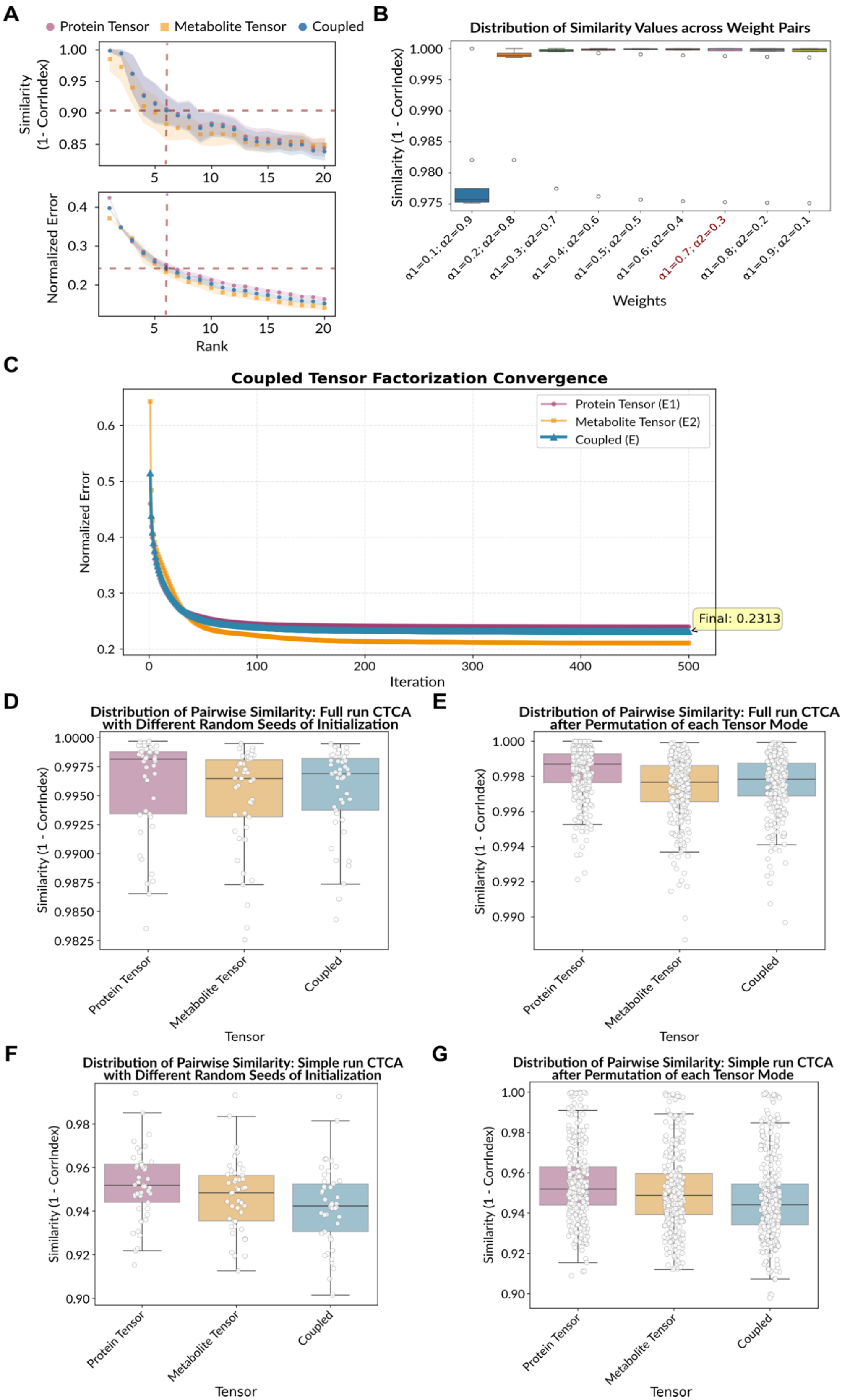

Supplementary Figure S2. Evaluation of CTCA performance, parameter selection, and robustness on single-cell cortical organoid data. (**A**) Selection of the number of factors (rank). The similarity of independent CTCA runs (top), computed as 1 - CorrIndex, where higher values indicate better reproducibility, and the normalized reconstruction error (bottom) are shown as a function of the number of factors, displayed per tensor (protein and metabolite-based) and for the coupled model. Shaded regions represent standard deviations across 20 runs per rank. The selected rank (red vertical dashed lines) balances high reproducibility (similarity over 0.9) and low reconstruction error. (**B**) Influence of balancing weights. Boxplots show the similarity across factorizations obtained using different balancing weight combinations (α1, α2) for the protein and metabolite tensors, respectively. The selected weights (α1 = 0.7; α2 = 0.3, highlighted in red) achieved the highest mean similarity with factorizations using other weight pairs,

providing a more representative model of all evaluated scenarios. (**C**) Convergence of the coupled tensor factorization. Loss represented as the normalized reconstruction error of the protein (*E1*) and metabolite (*E2*) tensors, and combined error for the coupled factorization (*E*) across iterations of the multiplicative update algorithm. The final error after reaching the stop criterion of 500 iterations is indicated. (**D**) Reproducibility across different model initializations. Distribution of pairwise similarities (1 - CorrIndex) among 10 independent full CTCA runs using different random seeds. Each full run was derived from 100 runs after best model selection, resulting in 1,000 total runs with unique seeds. (**E**) Robustness to input permutations. Distribution of pairwise similarities (1 - CorrIndex) among 25 independent full CTCA runs after permutations of elements in each tensor mode (five per mode). Each full run was derived from 100 runs after best model selection, resulting in 2,500 total runs. (**F**) and (**G**) are similar to (**D**) and (**E**), respectively, but with simple runs of the CTCA instead of the full runs (i.e., one run instead of the one hundred runs in each full run). As in (**C**), panels (**D**–**G**) are displayed separately for the protein and metabolite tensors, and the coupled models.

Supplementary Section S4: Biological evidence of potential coordination between protein and metabolite ligands

To provide evidence about whether protein- and metabolite-mediated CCC can be coordinated and, therefore, coupled for further analysis, we constructed a transcription factor (TF)-based regulatory network integrating external data sources. Specifically, we combined TF-target gene links from the CollecTRI database (Müller-Dott et al., 2023) with TF activity previously computed from scATAC-seq data of developing human brain cortex (Ziffra *et al.*, 2021) to retain only TFs active in cortical development. We then used the links between these TFs and (i) protein ligands present in our protein-based CCC tensor and (ii) enzymes producing metabolite ligands in the metabolite-based tensor, subsequently replacing enzymes with their corresponding metabolites to visualize and work with both modalities jointly. The resulting network (Supplementary Fig. S3A) reveals that several TFs regulate both protein and metabolite ligands (Supplementary Fig. S4A), suggesting shared upstream control across the two communication modalities.

To further assess whether our CTCA captures biologically meaningful patterns, we restricted the network to ligands with high CTCA loadings across all factors (Fig. 1F) and their cognate TFs, as shown in Supplementary Fig. S3B. We observe that this network leads to an enrichment for pairs of protein-metabolite ligands sharing TFs controlling their production, with an odds ratio of 1.5519 and P-value of 0.0121 after using Fisher's exact test. Moreover, we built factor-specific regulatory subnetworks including only ligands with high loadings within each factor, and we observed that every factor presented an odds ratio over 1.5 while most of them were significant (Supplementary Table S1). Factors 2 and 5 led to odds ratios of 4.0218 and 6.9226, respectively, suggesting that our CTCA can successfully identify CCC programs prioritizing coordination through, partly, transcriptional co-regulation of ligands from both modalities. By limiting the factor-specific networks to include only TFs co-regulating key ligands (Supplementary Fig. S5), we identified a list of TFs that could be important across these CCC programs and for cortical development (Supplementary Fig. S4). For example, ASCL1 is a TF that increased its ranking after filtering for high loading ligands (Supplementary Fig. S4B), which is known as a critical regulator of neuronal differentiation (Lundie-Brown *et al.*, 2025). Thus, our regulatory network analysis supports that coupled factorization can identify patterns of coordination between both CCC modalities in a biologically meaningful manner, which could be partially explained by a transcriptional co-regulation of protein- and enzymes producing metabolite ligands.

Finally, we evaluated whether receptors from both CCC modalities participate in related biological processes by performing a Gene Set Enrichment Analysis (GSEA) based on the ligand-receptor loadings per factor (Supplementary Fig. S6). For each factor, protein-based and metabolite-based loadings were independently max-scaled and concatenated, reflecting their importance within their modalities, to later create unified rankings that informed about their coordinated role. These LR pairs were then annotated with Gene Ontology (Biological Process) terms based on receptor genes. The resulting enrichments revealed processes shared between modalities that are also pertinent to cortical development, such as synapse processes in Factor 6, suggesting a coordination of functions between both types of communication.

Together, these results provide additional evidence that both protein- and metabolite-mediated CCC can be co-regulated through shared transcriptional programs and can share functions in the receiver cells, underscoring the biological relevance of the coupled factorization performed by Tensor-cell2cell v2. Nevertheless, it is important to highlight that multimodal CCC can be coordinated through other mechanisms that are not reflected at the level of the TFs-ligands regulatory network, or pathways in common where receptors of distinct CCC modalities are involved, which would require the integration of further data and analyses to gain insights about them.

Supplementary Table S1. Enrichment analysis of protein-metabolite ligand pairs controlled by shared transcription factors.

Factor	Odds Ratio	P-value	Adjusted P-value*
Factor 1	1.585153	0.088085	0.105702
Factor 2	4.021834	0.000189	0.000568
Factor 3	2.639485	0.027843	0.041764
Factor 4	1.752504	0.125655	0.125655
Factor 5	6.922645	0.000005	0.000027
Factor 6	1.698864	0.023908	0.041764

*P-values were adjusted across all factors using the Benjamini-Hochberg correction for multiple testing.

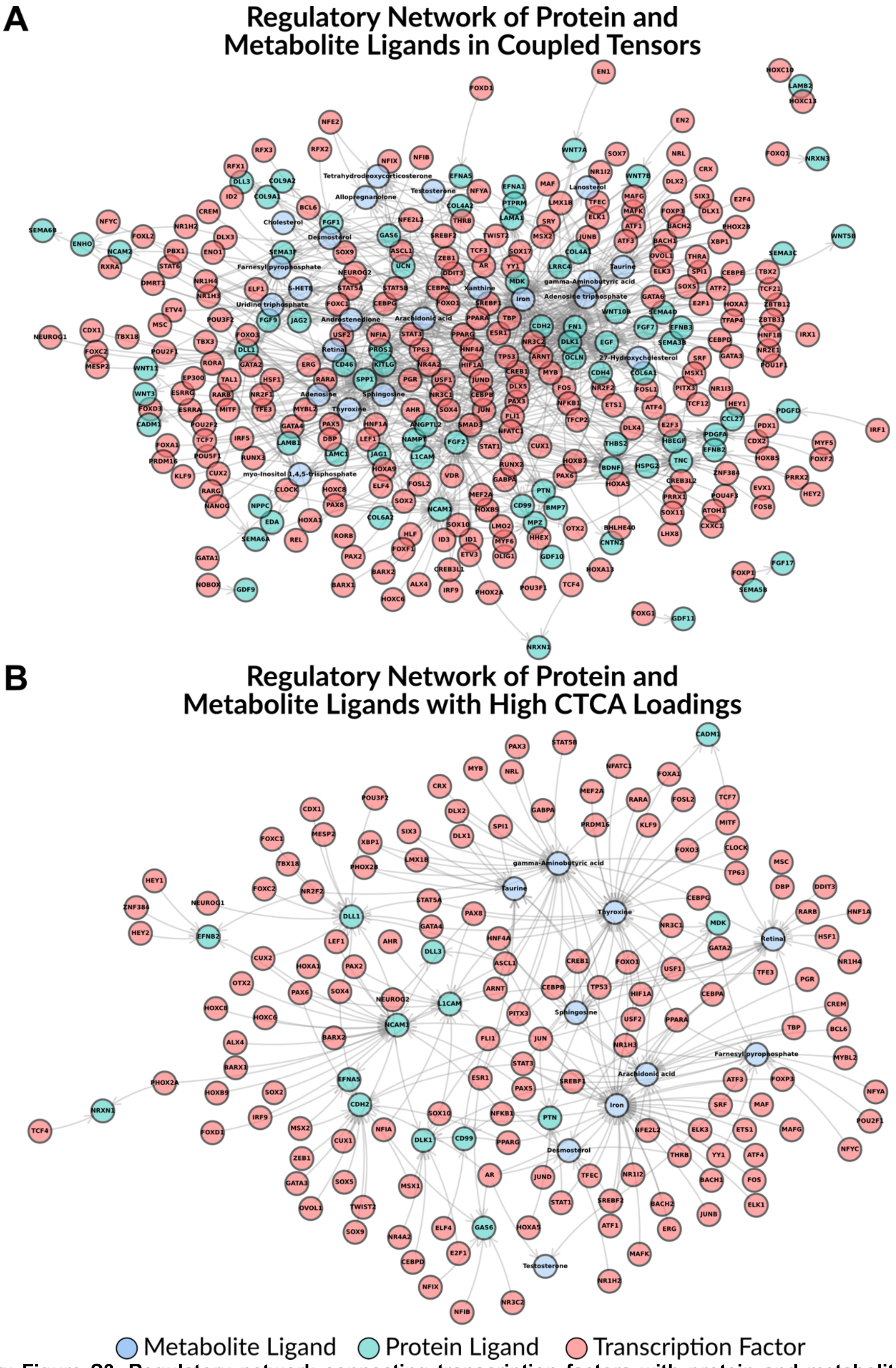

Supplementary Figure S3. Regulatory network connecting transcription factors with protein and metabolite ligands. (**A**) Regulatory network of transcription factors (TFs) linked to protein and metabolite ligands identified in the coupled tensors. This network was built from TF-target links collected from the CollecTRI database, retaining only TFs known to participate in cortical development (as determined from scATAC-seq data in (Ziffra *et al.*, 2021)). Initially, we connected TFs to protein ligands and enzymes producing metabolite ligands, but the latter were replaced by their corresponding metabolite ligands, allowing direct visualization of metabolite-based signals. (**B**) Filtered network including only ligands with high CTCA loadings (Fig. 1F) and their regulatory TFs. Nodes are colored as indicated in the legend at the bottom.

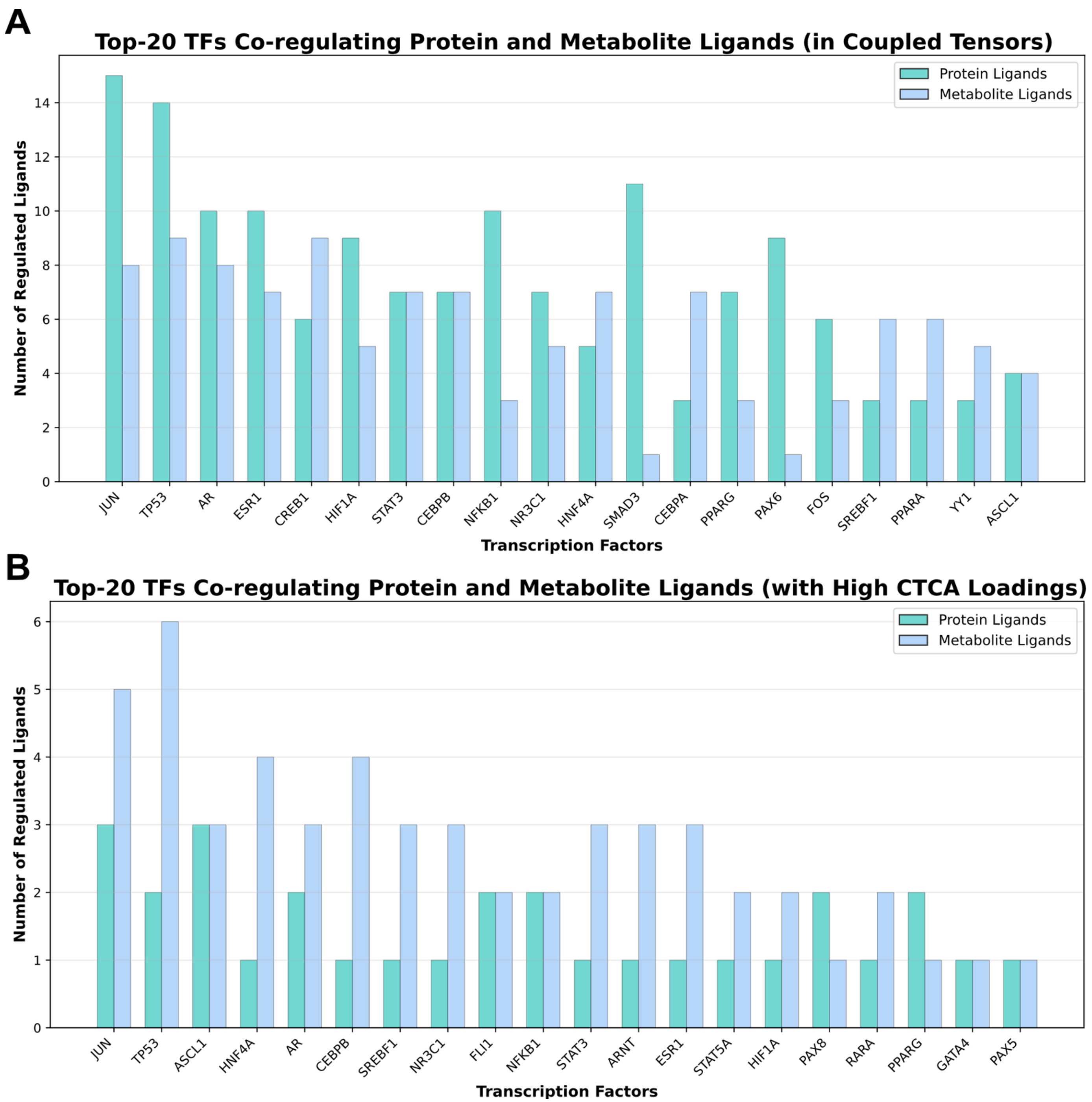

Supplementary Figure S4. Main transcription factors co-regulating protein and metabolite ligands. (**A**) Top 20 TFs co-regulating protein and metabolite ligands present in the coupled tensors, ranked by the number of connected ligands. **(B**) Same as (**A**), but limited to ligands with high CTCA loadings in Fig. 1F. Bars represent the number of protein and metabolite ligands regulated by each TF (colored as indicated in the legend). These top TFs are likely to be key co-regulators driving coordinated protein- and metabolite-mediated communication in cortical organoids.

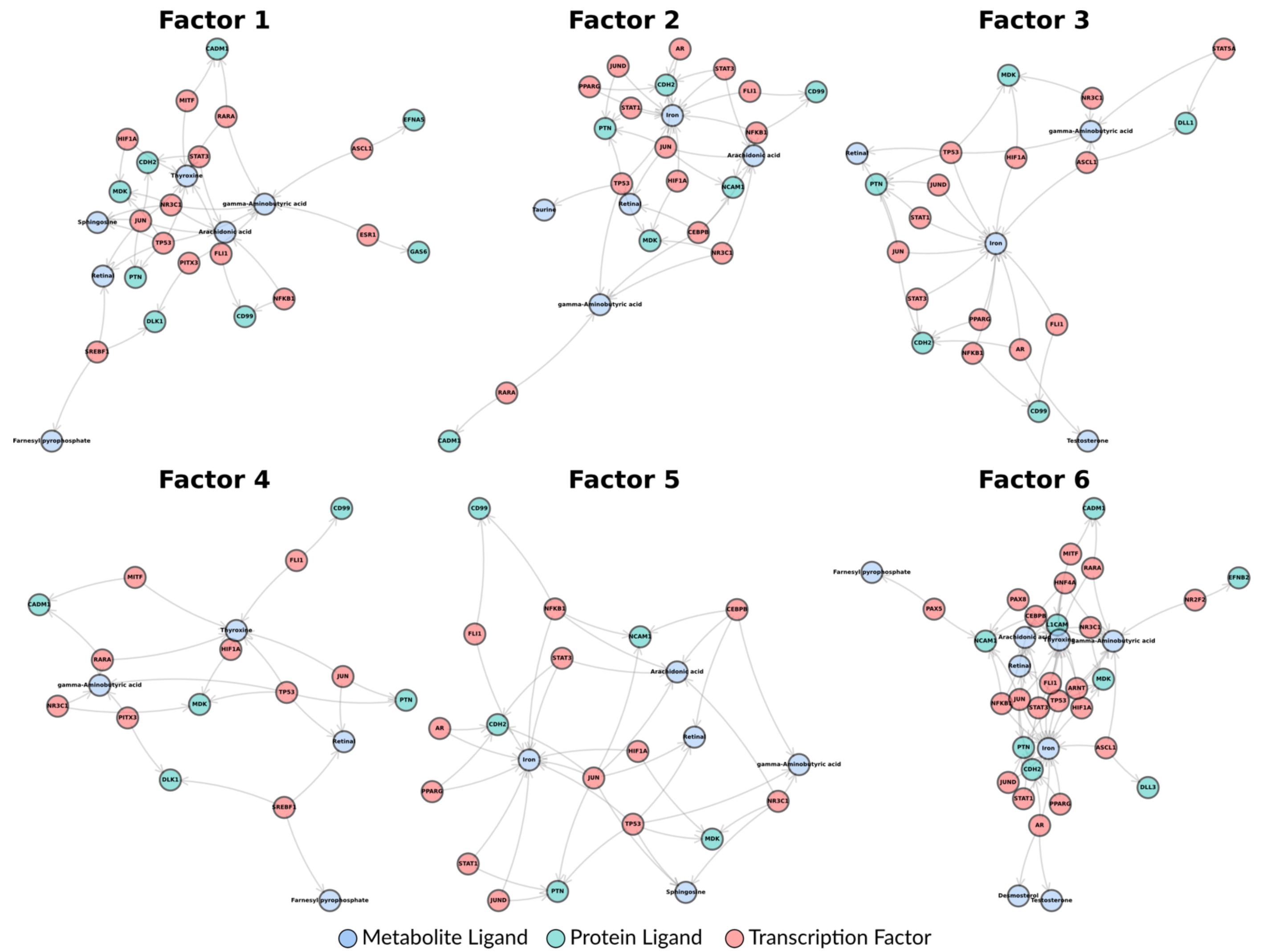

Supplementary Figure S5. Factor-specific regulatory networks resulting from the CTCA. Networks were constructed by including only ligands with high loadings within their modalities for each CTCA factor. In this case, only transcription factors (TFs) connecting both communication modalities are shown. Each panel corresponds to a distinct factor-specific subnetwork as indicated by the title, each of them showing TFs co-regulating protein and metabolite ligands. These subnetworks, built from the regulatory network in Supplementary Fig. S3, highlight factor-dependent regulatory programs potentially driving coordinated protein- and metabolite-mediated signaling.

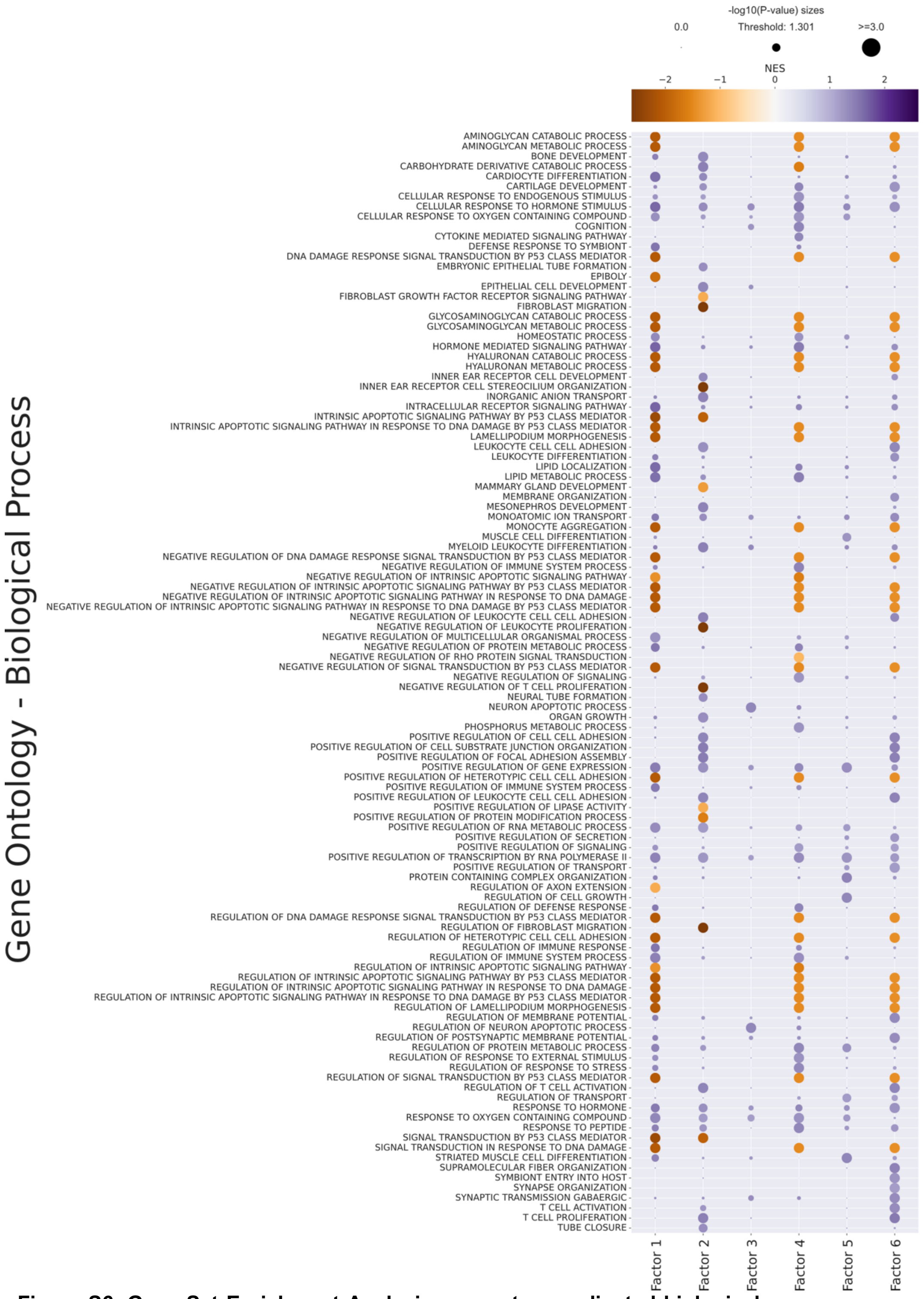

Supplementary Figure S6. Gene Set Enrichment Analysis suggests coordinated biological processes across protein and metabolite communication modalities. To assess whether receptors from both modalities within each factor participate in similar biological functions, we performed GSEA (Subramanian *et al.*, 2005) on the ligand-receptor pair loadings. For each factor, loadings for protein-based and metabolite-based LR pairs were independently max-scaled (to maximum value of one) and concatenated to create unified rankings across modalities. LR pairs were then annotated with Gene Ontology (Biological Process) gene sets based on their receptor genes. Then, GSEA was performed following the procedure described for Tensor-cell2cell v1 (Armingol *et al.*, 2022), applied here to the pre-ranked concatenated loadings from both modalities to evaluate whether receptors from both modalities within the same factor may be participating in similar biological processes, thereby suggesting biologically meaningful coordination. Dot sizes indicate significance levels, scaled proportionally to -$\log_{10}$(P-value); the threshold dot indicates the size of significance (P-value = 0.05). Dot colors represent the normalized enrichment score (NES) obtained from GSEA permutation testing, as shown in the colorbar. P-values were computed from 1,000 permutations and adjusted across all factors using the Benjamini-Hochberg correction for multiple testing.

Supplementary References

Armingol E, Baghdassarian HM, Martino C *et al.* Context-aware deconvolution of cell-cell communication with Tensor-cell2cell. *Nat Commun* 2022;**13**:3665.

Korsunsky I, Millard N, Fan J *et al.* Fast, sensitive and accurate integration of single-cell data with Harmony. *Nat Methods* 2019;**16**:1289–96.

Kossaifi J, Panagakis Y, Anandkumar A *et al.* TensorLy: Tensor Learning in Python. *arXiv [csLG]* 2016.

Li L, Yan S, Horner D *et al.* Revealing static and dynamic biomarkers from postprandial metabolomics data through coupled matrix and tensor factorizations. *Metabolomics* 2024;**20**:86.

Lundie-Brown J, Puletti F, Philpott A *et al.* Cell fate acquisition and reprogramming by the proneural transcription factor ASCL1. *Open Biol* 2025;**15**:250018.

Müller-Dott S, Tsirvouli E, Vazquez M *et al.* Expanding the coverage of regulons from high-confidence prior knowledge for accurate estimation of transcription factor activities. *Nucleic Acids Res* 2023;**51**:10934–49.

Rabanser S, Shchur O, Günnemann S. Introduction to tensor decompositions and their applications in machine learning. *arXiv [statML]* 2017.

Sobhani E, Comon P, Jutten C *et al.* CorrIndex: A permutation invariant performance index. *Signal Processing* 2022;**195**:108457.

Stuart T, Butler A, Hoffman P *et al.* Comprehensive integration of single-cell data. *Cell* 2019;**177**:1888–902.e21.

Subramanian A, Tamayo P, Mootha VK *et al.* Gene set enrichment analysis: a knowledge-based approach for interpreting genome-wide expression profiles. *Proc Natl Acad Sci U S A* 2005;**102**:15545–50.

Trujillo CA, Gao R, Negraes PD *et al.* Complex oscillatory waves emerging from cortical organoids model early human brain network development. *Cell Stem Cell* 2019;**25**:558–69.e7.

Williams AH, Kim TH, Wang F *et al.* Unsupervised discovery of demixed, low-dimensional neural dynamics across multiple timescales through tensor component analysis. *Neuron* 2018;**98**:1099–115.e8.

Ziffra RS, Kim CN, Ross JM *et al.* Single-cell epigenomics reveals mechanisms of human cortical development. *Nature* 2021;**598**:205–13.
